# Cdc42-Dependent Activation of NADPH Oxidase Is Involved in Ethanol-Induced Neuronal Oxidative Stress

**DOI:** 10.1371/journal.pone.0038075

**Published:** 2012-05-25

**Authors:** Xin Wang, Zunji Ke, Gang Chen, Mei Xu, Kimberly A. Bower, Jacqueline A. Frank, Zhuo Zhang, Xianglin Shi, Jia Luo

**Affiliations:** 1 Graduate Center for Toxicology, University of Kentucky, Lexington, Kentucky, United States of America; 2 Department of Internal Medicine, University of Kentucky, Lexington, Kentucky, United States of America; Virginia Commonwealth University, United States of America

## Abstract

It has been suggested that excessive reactive oxygen species (ROS) and oxidative stress play an important role in ethanol-induced damage to both the developing and mature central nervous system (CNS). The mechanisms underlying ethanol-induced neuronal ROS, however, remain unclear. In this study, we investigated the role of NADPH oxidase (NOX) in ethanol-induced ROS generation. We demonstrated that ethanol activated NOX and inhibition of NOX reduced ethanol-promoted ROS generation. Ethanol significantly increased the expression of p47^phox^ and p67^phox^, the essential subunits for NOX activation in cultured neuronal cells and the cerebral cortex of infant mice. Ethanol caused serine phosphorylation and membrane translocation of p47^phox^ and p67^phox^, which were prerequisites for NOX assembly and activation. Knocking down p47^phox^ with the small interfering RNA was sufficient to attenuate ethanol-induced ROS production and ameliorate ethanol-mediated oxidative damage, which is indicated by a decrease in protein oxidation and lipid peroxidation. Ethanol activated cell division cycle 42 (Cdc42) and overexpression of a dominant negative (DN) Cdc42 abrogate ethanol-induced NOX activation and ROS generation. These results suggest that Cdc42-dependent NOX activation mediates ethanol-induced oxidative damages to neurons.

## Introduction

Heavy alcohol consumption can cause structural and functional abnormalities of both the developing and mature brain [1], [2]. Alcohol exposure during development produces fetal alcohol spectrum disorders (FASD) which encompass a broad array of neuropathologic and systemic lesions as wells as neurocognitive and behavioral disabilities [3]. Prenatal alcohol exposure is recognized as the leading non-genetic cause of mental retardation in the Western World [4]. Binge alcohol exposure also induces neurodegeneration in the adult brain and is associated with neurocognitive deficits and Wernicke-Korsakoff syndrome [5], [6], [7].

Oxidative stress, which is caused by excessive production of reactive oxygen species (ROS), has been proposed as a potential mechanism for ethanol-induced neuronal damage [8], [9], [10], [11], [12]. Scavenging ROS are shown to protect neurons against ethanol neurotoxicity [13], [14]. However, the mechanisms underlying ethanol-induced ROS production remain unclear.

The NOX family NADPH oxidases, enzymes that transfer electrons across biological membranes, are identified as a major source of cellular ROS [15]. There are seven members of the NOX family: NOX1 through NOX5 and DuoX1/DuoX2. NOX2, also known as gp91^phox^, is the prototype NADPH oxidase. NOX enzymes are composed of catalytic and regulatory subunits, and their activation depends on the formation of an active complex with several cytosolic factors (p47^phox^, p67^phox^, and p40^phox^) which translocate to the membrane after activation [15]. NOX enzymes are ubiquitously expressed [15], [16]. Neurons and neutrophils predominantly express NOX2 [17]. Ethanol has been shown to up-regulate the expression NOX in many organs, including the lungs and liver, as well as in mouse embryos [18], [19], [20], suggesting that NOX may be involved in ethanol-induced ROS generation. We hypothesize that ethanol can activate NOX in neuronal cells, resulting in the accumulation of intracellular ROS. In this study, we investigated the effect of ethanol on NOX activation. Furthermore, we demonstrated the involvement of cell division cycle 42 (Cdc42), a Rho-family GTP-binding protein (GTPase), in ethanol-induced NOX activation and ROS generation.

## Materials and Methods

### Reagents

Protein G-Sepharose beads were purchased from Amersham Biosciences (Pittsburgh, PA). Antibodies were purchased from Santa Cruz (Santa Cruz, CA). Lipofectamine 2000 reagent was purchased from Invitrogen Corporation (Carlsbad, CA). Other chemicals were purchased from Sigma Chemical Co. (St. Louis, MO) unless otherwise mentioned.

**Figure 1:**
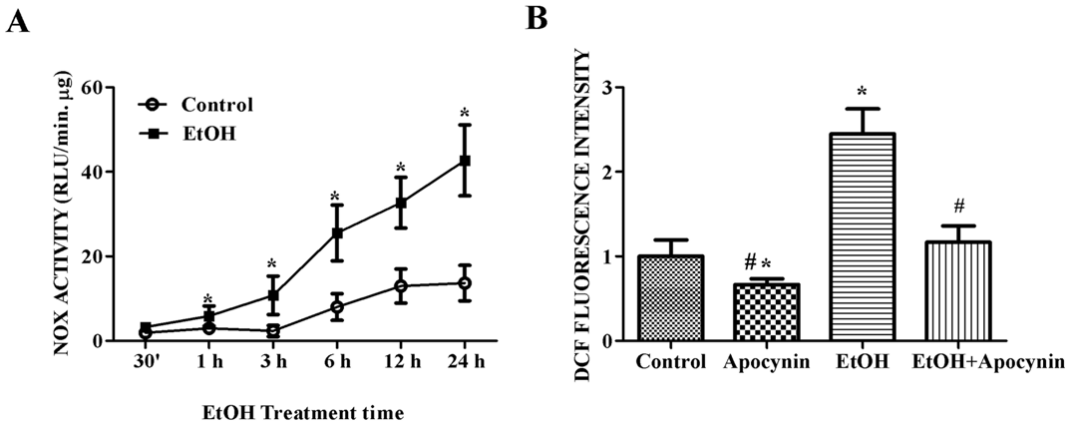
Effect of ethanol on NOX activation and ROS generation. **A**. SH-SY5Y cells were exposed to ethanol (0 or 0.4%) for indicated times. NOX activity was determined by lucigenin chemiluminescence assay as described under the Materials and Methods. **B**. SH-SY5Y cells were pretreated with apocynin (0 or 50 μM) for 2 hours followed by exposure to 0.4% ethanol for 24 hours. Cells were labeled with DCFDA (10 μM) as described under the Materials and Methods and relative intensity of DCFDA (cellular ROS concentration) was measured and presented. The data are expressed as the mean ± SEM of three independent experiments. * denotes statistically significant difference from control cells (*p*<0.05); # denotes statistically significant difference from ethanol-treated cells (*p*<0.05).

### Cell culture and ethanol exposure

Human neuroblastoma cells, SH-SY5Y, were obtained from American Type Culture Collection (ATCC, Manassas, VA) and cultured in Dulbecco's modified Eagle's medium (DMEM) supplemented with 10% fetal bovine serum (FBS), 2 mM L-glutamine, and 100 U/ml penicillin and 100 µg/ml streptomycin at 37°C with 5% CO_2_. A method utilizing sealed containers was used to maintain ethanol concentrations in the culture medium. With this method, ethanol concentrations in the culture medium can be accurately maintained [21]. A pharmacologically relevant concentration of 0.4% was used in this study. In general, the concentration for *in vitro* studies is higher than that required to produce a similar effect *in vivo* [21].

**Figure 2:**
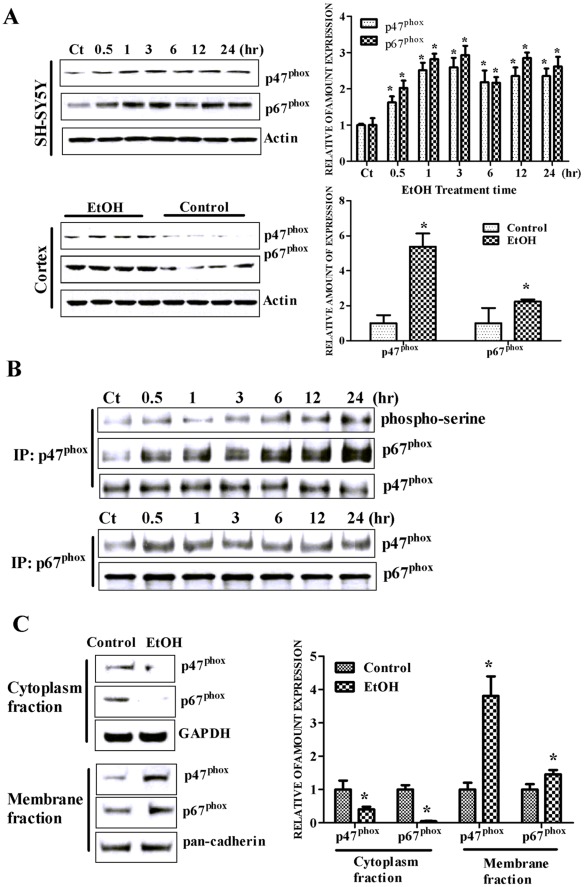
Effect of ethanol on p47^phox^ and p67^phox^. **A**. Top: SH-SY5Y cells were treated with ethanol (0 or 0.4%) for specified times. The expression of p47^phox^ and p67^phox^ was determined by immunoblotting. The expression of actin served as an internal loading control. Relative amounts of p47^phox^ and p67^phox^ was measured by densitometry and normalized to the expression of actin. Bottom: 7-days-old mice were exposed to ethanol as described under the Materials and Methods. The expression of p47^phox^ and p67^phox^ in the prefrontal cortex was determined with immunoblotting and quantified as described above. The experiment was replicated three times. The data are expressed as the mean ± SEM of three independent experiments. * denotes statistically significant difference from control group (*p*<0.05). **B**. SH-SY5Y cells were exposed to ethanol (0 or 0.4%) for specified times. After the treatment, cells were lysed and the lysates were immunoprecipitated (IP) with an anti-p47^phox^ or anti-p67^phox^ antibody. The immunocomplexes were resolved by electrophoresis on a 10% SDS-polyacrylamide gel followed by immunoblotting analysis using either an anti-phosphoserine, anti-p47^phox^, or anti-p67^phox^ antibody. **C**. Cytoplasm and membrane proteins were extracted as described under the Materials and Methods. The expression of p47^phox^ and p67^phox^ in the cytoplasm/membrane fractions was determined with immunoblotting. The expression of GAPDH and pan-cadherin served as internal loading controls for cytoplasmic and membrane fractions, respectively. Relative amounts of p47^phox^ and p67^phox^ was measured by densitometry and normalized to the expression of GAPDH or pan-cadherin. The experiment was replicated three times. The data are expressed as the mean ± SEM of three independent experiments. * denotes statistically significant difference from control group (*p*<0.05).

### ROS detection

Cellular ROS was determined using the fluorescent dye DCFDA as described previously [22]. DCFDA is oxidized by hydrogen peroxide (H_2_O_2_), peroxynitrite, or hydroxyl radical into a fluorescent DCF signal. SH-SY5Y cells were plated in six-well plates and exposed to ethanol. After treatment, the cells were incubated with CM-H_2_DCFDA (10 µM) in the dark for 30 min at 37°C. Intracellular ROS levels (DCF signals) were measured with a flow cytometer (FACSAria^TM^, BD Biosciences, USA) at an excitation wavelength of 492 nm and an emission wavelength of 517 nm.

### Animals and ethanol exposure

C57BL/6 mice were obtained from Harlan Laboratories (Indianapolis, IN) and maintained at the Animal Facility of the University of Kentucky Medical Center. All procedures were performed in accordance with the guidelines set by the NIH and the Animal Care and Use Committee of the University of Kentucky. The Institutional Animal Care & Use Committee (IACUC) has specifically approved this study. An acute ethanol exposure paradigm, which had been shown to induce robust neurodegeneration in infant mice, was employed [23]. Briefly, each 7-day-old mouse pup in a litter was assigned to either a control (saline) or ethanol group. The mice were injected subcutaneously with saline or ethanol (2.5 g/kg, 20% solution in saline) twice at 0 hr and 2 hr. Eight hours after the first ethanol injection, the brains were removed and processed for immunoblotting analysis. The blood ethanol concentration was 338.8±26.5 mg/dl at 8 hours after the first ethanol injection [24].

**Figure 3:**
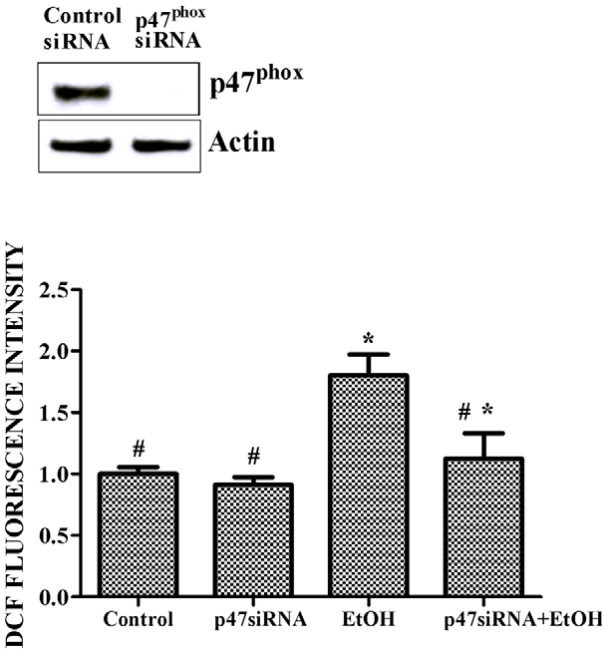
Effect of p47^phox^ siRNA on ethanol-induced ROS generation. SH-SY5Y cells were transfected with p47^phox^ siRNA for 48 hours as described under the Materials and Methods. The expression of p47^phox^ was determined by immunoblotting (top panel). SH-SY5Y cells were transfected with p47^phox^ siRNA for 48 hours and exposed to ethanol (0 or 0.4%) for 24 hours. The relative amount of ROS was measured as described above (bottom panel). The data are expressed as the mean ± SEM of three independent experiments. * denotes statistically significant difference from control cells (*p*<0.05); # denotes statistically significant difference from ethanol-treated cells (*p*<0.05).

### Immunoprecipitation and immunoblotting analysis

The procedure for immunoprecipitation and immunoblotting has been previously described [22]. Briefly, for immunoprecipitation, an aliquot of cell lysates containing 200 µg of proteins were pre-cleared with protein G-Sepharose beads (Amersham Biosciences, USA). The unbounded proteins were incubated with either anti-p47^phox^ or anti-p67^phox^ antibody (1∶50) overnight at 4°C. Protein G-Sepharose was then added and mixed at 4°C for 3 hours. Immunoprecipitates were collected by centrifugation at 10,000 g for 10 min. After washing four times with PBS, the pellets were resuspended in 20 µL 2× sodium dodecyl sulfate sample buffer and analyzed with immunoblotting. The activation of p47^phox^ by serine phosphorylation was detected by immunoblotting analysis using an anti-phosphoserine antibody. Protein samples were loaded into the lanes of a sodium dodecyl sulfate-polyacrylamide gel. The proteins were separated by electrophoresis and transferred to nitrocellulose membranes. The membranes were blocked with 5% nonfat dry milk in 0.01 M Tris-buffered saline (TBS) (pH 7.4) and 0.05% Tween-20 (TBST) at room temperature 1 hour. Subsequently, the membrane was incubated with primary antibodies directed against target proteins overnight at 4°C. The final dilution for primary antibodies was: p47^phox^ (1∶1,000), p67^phox^ (1∶1,000), phosphoserine (1∶1,000), Cdc42, (1∶1,000) and actin (1∶3,000). After two quick washes in TBST, the membranes were incubated with secondary antibodies conjugated to horseradish peroxidase (Amersham, USA) diluted at 1∶5,000 in TBST for 1 hour. The immunocomplexes were detected by the enhanced chemiluminescence method (Amersham). The intensity of protein expression was quantified with the software of Image J (Version 1.42, National Institutes of Health).

**Figure 4:**
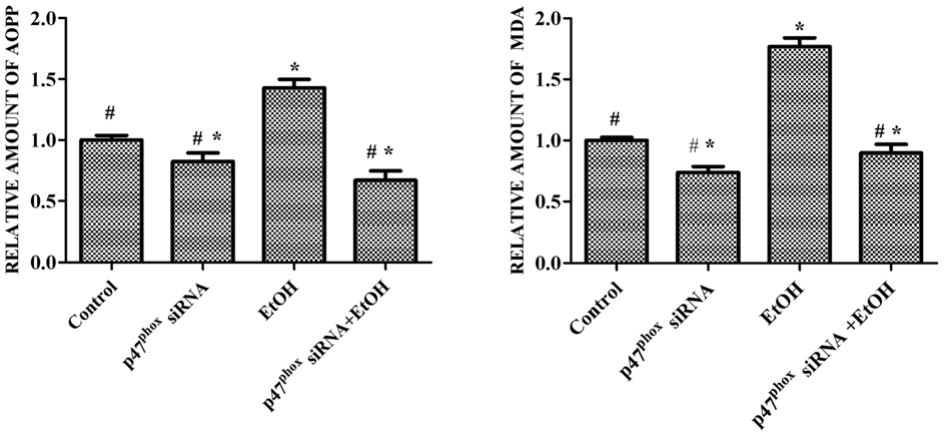
Effect of p47^phox^ siRNA on ethanol-induced oxidative damage. SH-SY5Y cells were transfected with p47^phox^ siRNA for 48 hours and treated with ethanol (0 or 0.4%) for 48 hours. Protein oxidation and lipid peroxidation were determined by AOPP and MDA assay, respectively as described under the Materials and Methods. The data are expressed as the mean ± SEM of three independent experiments. * denotes statistically significant difference from control cells (*p*<0.05); # denotes statistically significant difference from ethanol-treated cells (*p*<0.05).

### Cell transfection

Dominant negative Cdc42 construct (phEFdnCdc42N14) was a gift from Dr. Yong Qian (National Institute for Occupational Safety and Health, Morgantown, WV). p47^phox^ siRNA was obtained from Santa Cruz (Santa Cruz, CA). SH-SY5Y cells were transfected with dominant negative Cdc42 or p47^phox^ siRNA using Lipofectamine 2000 reagent according to the manufacturer's instruction. Forty eight hours after transfection, cells were exposed to ethanol.

**Figure 5:**
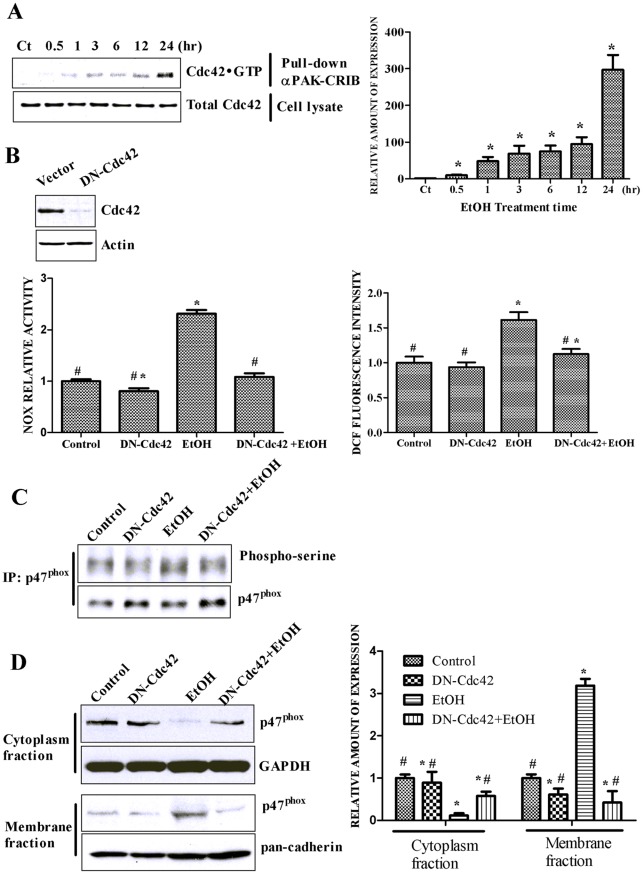
Role of Cdc42 in ethanol-induced NOX activation and ROS generation. **A**: SH-SY5Y cells were exposed to ethanol (0 or 0.4%) for indicated times. Active Cdc42 was pulled down by PAK1-PBD color agarose beads and analyzed by immunoblotting using an anti-Cdc42 antibody as described under the Materials and Methods (top panel). Relative expression of active Cdc42 was quantified (bottom panel). The data are expressed as the mean ± SEM of three independent experiments. * denotes statistically significant difference from control cells (*p*<0.05). **B**. SH-SY5Y cells were transfected with dominant-negative Cdc42 (DN-Cdc42) for 48 hours to inhibit endogenous Cdc42 activity. Active Cdc42 was determined as described above (top panel). After transfection with DN-Cdc42, cells were exposed to ethanol (0.4%, 24 hours). The effect of ethanol on NOX activity and ROS generation was investigated as described in Figure 1 (bottom panel). The data are expressed as the mean ± SEM of three independent experiments. * denotes statistically significant difference from control cells (*p*<0.05); # denotes statistically significant difference from ethanol-treated cells (*p*<0.05). **C**. SH-SY5Y cells were transfected with DN-Cdc42 for 48 hours. After transfection, cells were exposed to ethanol (0.4%, 24 hours). The effect of ethanol on p47^phox^ phosphorylation was investigated as described in Figure 2B. **D**. SH-SY5Y cells were transfected with DN-Cdc42 for 48 hours. After transfection with DN-Cdc42, cells were exposed to ethanol (0.4%, 24 hours). Cytoplasm and membrane proteins were extracted as described under the Materials and Methods. The expression of p47^phox^ in the cytoplasm/membrane fractions was determined with immunoblotting. The expression of GAPDH and pan-cadherin served as internal loading controls for cytoplasmic and membrane fractions, respectively. Relative amounts of p47^phox^ was measured by densitometry and normalized to the expression of GAPDH or pan-cadherin. The experiment was replicated three times. The data are expressed as the mean ± SEM of three independent experiments. * denotes statistically significant difference from control cells (*p*<0.05). # denotes statistically significant difference from ethanol-treated cells (*p*<0.05).

### Measurement of malondialdehyde (MDA)

MDA is formed during lipid peroxidation. Ethanol-induced lipid peroxidation was assessed by the measurement of MDA using a commercial kit (Cell Biolab, San Diego) according to the manufacturer's instruction. Briefly, the cells were lysed by repetitive freeze-thawing and were mixed with N-methyl-2-phenylindole in acetonitrile and methanol for 2 hours at 95°C. The samples were then centrifuged at 4,000 g for 10 min. The MDA in the supernatant was determined with a spectrophotometer at the wavelength of 532 nm.

### Cdc42-GTP binding assay

Cdc42 activation assays were performed by a pull-down assay using PAK1-PBD color agarose beads (Cell Biolabs, USA). Briefly, 300 µg protein samples were mixed with 20 µl of PAK1-PBD agarose beads and incubated for 1 hour at 4°C. The reaction was terminated by the addition of MgCl_2_. The agarose beads were collected by spinning at 12,000 g for 1 min at 4°C, and the supernatant were removed. Precipitated complexes were washed and subjected to immunoblotting analysis using an anti-Cdc42 antibody.

### Isolation of membrane and cytosolic fractions

Membrane and cytosolic fractions were separated by centrifugation as previously described [22]. Briefly, cells were harvested with cell dissociation solution (PBS-EDTA), washed with ice-cold PBS, and centrifuged for 5 min at 700 g. Supernatants were discarded, and the pellets were resuspended in ice-cold Tris-sucrose buffer containing 10 mM Trizma base, 340 mM sucrose, 1 mM EGTA, and 10 μg/ml protease inhibitor mixture at pH 7.1. The pellets were sonicated by four 15-second bursts. The cellular homogenates were clarified by centrifugation at 1,475 g at 4°C to remove nuclei and unbroken cells. The supernatant was then ultracentrifuged at 100,000 g for 1 hour at 4°C. The pellet, which was referred to as the membrane fraction, was resuspended in Tris-sucrose buffer, and stored at −80°C. The supernatant was referred to as the cytosolic fraction.

### NOX activity assay

NOX activity was measured by the lucigenin-enhanced chemiluminescence method as described [22]. Briefly, cultured cells were homogenized in lysis buffer (20 mM KH_2_PO4, pH 7.0, 1 mM EGTA, 1 mM phenylmethylsulfonyl fluoride, 10 μg/ml aprotinin, and 0.5 μg/ml leupeptin) by using a Dounce homogenizer (100 strokes on ice). Homogenates were centrifuged at 800 *g* at 4°C for 10 min to remove the unbroken cells and debris. For determining NOX activity, 100 μl aliquots of homogenates were added to 900 μl of 50 mM phosphate buffer containing 1 mM EGTA, 150 mM sucrose, 5 μM lucigenin, and 100 μM NADPH, pH 7.0. Photon emission was measured in a luminometer every 30 seconds for 5 min. There was no measurable activity in the absence of NADPH. Protein concentration was determined using the Bio-Rad protein assay reagent. Superoxide anion production was expressed as relative chemiluminescence (light) units (RLU)/mg protein.

### Measurement of advanced oxidation protein products (AOPP)

Protein oxidation was assessed by the measurement of AOPP using a commercial kit (Cell Biolab, USA). Briefly, ten microliters of acetic acid was added to two 200 µl of protein sample or standard. After incubation for 5 min at room temperature, the reaction was terminated by adding 20 µl of potassium iodide. AOPP were measured by spectrophotometry at the absorbance of 340 nm and calibrated with chloramine-T solutions. AOPP concentration (micromoles per liter of chloramine-T equivalents) was calculated and expressed relative to untreated controls.

### Statistical analysis

Differences among treatment groups were tested using ANOVA. Differences in which *p* was <0.05 were considered statistically significant. In cases where significant differences were detected, specific *post hoc* comparisons between treatment groups were examined with Student-Newman-Keuls tests. The analyses were performed using SPSS software (SPSS, Chicago, IL, USA).

## Results

### Ethanol activates NADPH oxidase (NOX) and induces NOX-dependent ROS generation

To determine whether NOX is involved in ethanol-induced neuronal oxidative stress, we first examined the effect of ethanol on NADPH oxidase activity *in vitro*. As shown in Fig. 1A, ethanol (0.4%) caused a duration-dependent increase in NADPH oxidase activity in SH-SY5Y cells. The ethanol-induced increase in NOX activity was statistically significant after one hour of exposure. After 24 hours of exposure, ethanol increased NOX activity by 3.1 fold. Exposure to 0.4% ethanol for 24 hours significantly increased the production of ROS which was indicated by a 2.4-fold increase of DCF fluorescence intensity (Fig. 1B). Treatment of apocynin, a NOX inhibitor, not only decreased basal DCF fluorescence intensity, but also blocked ethanol-induced ROS production. The result suggested that NOX was involved in ethanol-stimulated ROS production.

### p47^phox^ mediates ethanol-induced NOX activation

p47^phox^ and p67^phox^ are critical subunits for NOX activation. We examined the effect of ethanol on the expression of p47^phox^ and p67^phox^. As shown in Fig. 2A, ethanol significantly increased the expression of p47^phox^ and p67^phox^ in SH-SY5Y cells in a duration-dependent manner. The peak increase was observed at 3 hours after ethanol exposure; ethanol increased the expression of p47^phox^ and p67^phox^ by 2.5 fold and 2.8 fold following 3 hours of ethanol exposure, respectively. Similarly, ethanol up-regulated p47^phox^ and p67^phox^ expression in the developing brain. Ethanol exposure increased p47^phox^ and p67^phox^ expression in the cerebral cortex of 7-day-old mice by 5.3 fold and 2.2 fold, respectively (Fig. 2A).

Serine phosphorylation of p47^phox^ is a critical step for the cytoplasmic complex formation (p40^phox^-p47^phox^-p67^phox^) and an indication of NOX activation [25], [26]. We therefore examined the effect of ethanol on serine phosphorylation of p47^phox^. In this experiment, p47^phox^ was immunoprecipitated with an anti-p47^phox^ antibody and probed with an anti-phosphoserine antibody to detect serine phosphorylation of p47^phox^. Our results indicated that ethanol not only increased serine phosphorylation of p47^phox^, but also promoted the formation of a complex with p67^phox^ (Fig. 2B). The translocation of p47^phox^ and p67^phox^ is also an indication of NOX activation. We examined the effect of ethanol on the distribution of p47^phox^ and p67^phox^. As shown in Fig. 2C, only a small amount of p47^phox^ and p67^phox^ was found in the membrane fraction of control cells. Ethanol exposure (0.4%, 6 hours) increased the membrane-associated p47^phox^ and p67^phox^ by 3.8 fold and 1.5 fold, respectively. At the same time, the levels of p47^phox^ and p67^phox^ in the cytoplasm fraction were decreased by ethanol exposure (Fig. 2C). Taken together, these results indicated that ethanol activated NOX by stimulating p47^phox^.

To determine the role of p47^phox^ in ethanol-induced ROS generation, we used RAN interference to down-regulate p47^phox^ and inhibit NOX activation. As shown in Fig. 3, transfection with p47^phox^ siRNA significantly mitigated ethanol-induced ROS generation.

### Down-regulation of p47^phox^ ameliorates ethanol-induced oxidative damage

Excessive free radicals can damage proteins, lipids, carbohydrates, and nucleic acids. Advanced oxidation protein products (AOPP) is a marker protein for oxidation and is commonly used as an indicator of oxidative damage [27]. Malondialdehyde (MDA) is formed by fatty acids with two or more double bonds and is used as a measure of lipid peroxidation. As shown in Fig. 4, ethanol exposure (48 hr, 0.4%) significantly increased the levels of AOPP and MDA in SH-SY5Y cells. Knocking-down p47^phox^ with siRNA blocked the ethanol-induced increase in AOPP and MDA. Therefore, inhibition of NOX activation by suppressing p47^phox^ was sufficient to ameliorate ethanol-induced oxidative damage.

### Cdc42 is involved in ethanol-induced NOX activity

We next investigated how ethanol activated NADPH oxidase. We previously showed that the activation of Cdc42 was involved in ethanol-induced ROS production in mouse endothelial (SVEC-10) cells [28]. It has been suggested the Rho family of small GTPases are involved in NOX activation [29]. Arsenic-induced NOX activation is mediated by Cdc42, but not Rac in endothelial cells [30]. We therefore hypothesized that Cdc42 regulated ethanol-induced NOX activation in SH-SY5Y cells. We first examined the effect of ethanol on Cdc42 activation in SH-SY5Y cells. As shown in Fig. 5A, ethanol-induced activation of Cdc42 was observed as early as one hour following ethanol exposure. By 24 hours after ethanol exposure, Cdc42 activity was increased by more than 300 fold. Expression of a dominant-negative (DN) Cdc42 in SH-SY5Y cells blocked ethanol-stimulated NOX activity and ROS generation (Fig. 5B). Furthermore, the DN-Cdc42 inhibited ethanol-induced phosphorylation and menbrane translocation of p47^phox^ (Figs. 5C and D). These results indicated that Cdc42 played a critical role in ethanol-induced NOX activation and ROS generation.

## Discussion

ROS is a collective term for the intermediates formed during oxidative metabolism, encompassing both oxygen radicals and non-radical reactive oxygen derivatives including superoxide anion (O_2_^−^·), the hydroxyl radicals (OH), and hydrogen peroxide (H_2_O_2_). Excessive ROS production has been suggested as an important cause of alcohol-induced brain damage [31]. ROS may be produced by multiple processes, such as mitochondrial electron transport chain and the activity of NOX, nitric oxide synthase, and xanthine oxidase [32]. Recent studies identify NOX as an important source of ROS production in various cells and tissues, including neurons and the brain [15], [33].

Our results indicate that NOX is an important mediator of ethanol-induced ROS generation in neuronal cells. However, we do not exclude the contribution of other pathways in other model systems. For example, it was reported that ethanol generates ROS and nitric oxide (NO) via induction of NOX/xanthine oxidase (XOX) and nitric oxide synthase (NOS) in human neurons [34]. Ethanol stimulates ROS generation by mitochondria through Ca^2+^ mobilization in hippocampal astrocytes [35]. The results reported in this study are obtained from ethanol treatment at a concentration of 0.4% (400 mg/dl). Ethanol at 0.2% produces a similar effect (data not shown), but with 0.4% ethanol, the outcomes are more robust and consistent. In general, the concentration for *in vitro* studies is higher than that required to produce a similar effect *in vivo* and 0.4% is a relevant concentration for *in vitro* study [21]. Ethanol also increased the expression of p47^phox^, and p67^phox^ in the developing brain. It is has been demonstrated that ethanol but not its metabolites is a causative agent for ethanol-induced damage to the developing brain [36]. It is therefore likely that the alteration in NOX is caused by ethanol but not its metabolites.

The NOX family has seven members, NOX1 through NOX5 and DuoX1/DuoX2 [17], [37]. The expression and function of NOX are cell- or tissue-specific. In neurons, the predominant form of NOX is NOX2 [17]. NOX2 consists of membrane-bound subunits, gp91^phox^ and p22^phox^, which form the flavocytochrome *b*_558_ complex together with the cytosolic subunits p40^phox^, p47^phox^, and p67^phox^ as well as small GTPase, Rac. Superoxide production is induced by the assembly of cytosolic and membrane-bound subunits, which is mediated through serine phosphorylation of p47^phox^ [38]. It has been demonstrated that the kinetics of serine phosphorylation of p47^phox^ paralleled that of NOX activation [38]. We find that ethanol increased the expression of p47^phox^ and p67^phox^ in cultured neuronal cells and in the developing brain. Furthermore, ethanol enhances serine phosphorylation of p47^phox^ as well as the translocation of p47^phox^ and p67^phox^ from the cytoplasm to the membrane, indicating NOX activation. Down-regulation of p47^phox^ is sufficient to attenuate ethanol-induced ROS production and oxidative damage, confirming that the activation of p47^phox^/NOX is indeed involved in ethanol-mediated ROS generation in neurons.

It has been suggested the Rho family of small GTPases are involved in NOX activation [29]. We have previously demonstrated that Cdc42, a member of the Rho family, mediates arsenic-induced NOX activation and ROS generation in endothelial cells independent of Rac [30]. We show here Cdc42 is activated by ethanol, and blockage of Cdc42 is sufficient to ameliorate ethanol-induced ROS production and oxidative damage; this suggests the same pathway (Cdc42/NOX) is involved in ethanol-induced ROS production. It is unclear how Cdc42 mediates NOX activation. It is suggested in endothelial cells that Cdc42-dependent activation of NOX is mediated by the rearrangement of actin filaments [30]. This possibility remains to be addressed in neurons.

In summary, the current study identifies a *novel* pathway for ethanol-induced ROS production in neurons and opens a new avenue for investigating the mechanisms of ethanol-induced damage to the brain. For example, using p47^phox^ deficient mice to evaluate ethanol-induced neurodegeneration and behavioral abnormality will not only validate the current finding, but also establish potential targets for therapeutic approaches.
